# A retrospective study of the antimicrobial susceptibility patterns of *Klebsiella pneumoniae* isolated from urine samples over a decade in South India

**DOI:** 10.3389/fmicb.2025.1553943

**Published:** 2025-06-17

**Authors:** Mahadevaiah Neelambike Sumana, Yogeesh D. Maheshwarappa, Kalyani G., Rashmi P. Mahale, Sowmya G. S, Morubagal Raghavendra Rao, Ranjitha Shankaregowda, Vidyavathi B. Chitaragi, Deepashree R., Sujatha S. R., Neetha S. Murthy, Badveti Satyasai, Vasimalli Vinay Kumar, Supreeta R. Shettar

**Affiliations:** ^1^Department of Microbiology, JSS Medical College and Hospital, JSS Academy of Higher Education and Research, Mysuru, India; ^2^IDepartment of General Pathology and Microbiology, Dayanand Sagar College of Dental Science, Bengaluru, India

**Keywords:** *Klebsiella pneumoniae*, carbapenem resistant *Klebsiella pneumoniae*, urinary tract infection, antimicrobial susceptibility patterns, multidrug resistant organism, antimicrobial resistance, antibiotic policies

## Abstract

**Introduction:**

Urinary tract infections (UTIs) are common bacterial infections with significant health implications. This study aimed to assess the antimicrobial susceptibility (AST) patterns of *Klebsiella pneumoniae (KPN)* isolated from urine samples over a decade (2014–2023).

**Methods:**

The study analyzed *Klebsiella pneumoniae* isolates AST pattern from urine samples at a tertiary care hospital in Mysuru (Karnataka), South India using the VITEK-2 (bioMérieux, France) database.

**Results:**

Of 73,283 urine samples, 21,362 (29.15%) showed significant bacterial growth. The most frequently isolated organism was *Escherichia coli* (*n* = 9,211, 43.11%), followed by *Pseudomonas aeruginosa* (*n* = 1,108, 5.18%) and *K. pneumoniae* (*n* = 920, 4.30%). Of the 920 *K. pneumoniae* isolates, 385 (41.84%) were carbapenem-resistant (CRKP). Isolation rates were higher in males (*n* = 229, 59.48%) than females (*n* = 156, 40.52%), with a statistically significant *p*-value (<0.0001). Carbapenem resistance rose from 3.70% in 2014 to 66.13% in 2021, then declined to 38.55% in 2023. Resistance to fluoroquinolones, aminoglycosides, and cephalosporins increased, with cephalosporin resistance exceeding 85% by 2023. These trends reflect growing drug resistance among *K. pneumoniae*.

**Conclusion:**

The study reveals a significant rise in antimicrobial resistance in Klebsiella pneumoniae, particularly to carbapenems and fluoroquinolones. Effective treatment requires antibiotic stewardship, strict infection control, and ongoing surveillance to maintain therapeutic options.

## 1 Introduction

Urinary Tract Infections (UTIs) are known to cause significant health issues, including complications such as strictures, abscesses, fistulas, pyelonephritis, bacteremia, and kidney dysfunction (Schwenger et al., 2015). Recent advancements in understanding the normal flora in the urinary tract have unveiled the urinary microbiome, consisting of various species (Neugent et al., 2020). Gram-negative bacteria often cause UTIs and present a major public health challenge due to their ability to acquire antibiotic resistance, compounded by the diminishing prospects of discovering new antibiotics (Peleg and Hooper, 2010). The mortality rates associated with UTIs, particularly from complications like pyelonephritis, underscore their severity, with rates reported as high as 1% in men and 3% in women (Peleg and Hooper, 2010). Approximately one in two women experience UTIs at some point in their lives, while the incidence in men varies with age, with higher rates after 50 years due to prostate enlargement. Additionally, elderly individuals are more susceptible to asymptomatic UTIs, with prevalence rates of 30% in women and 10% in men annually (Schwenger et al., 2015).

The treatment of UTIs is often challenging due to potential misinterpretation of infection severity by clinicians. Inadequate treatment resulting from underestimation, as well as excessive treatment due to overestimation, can lead to treatment failures and contribute to the growing problem of antimicrobial resistance. This underscores the critical need for accurate diagnostic approaches and judicious antibiotic use to mitigate the emergence of resistant strains (Blondeau, 2004). UTIs are predominantly caused by bacteria, with uropathogenic *Escherichia coli* (UPEC) being the most common pathogen, followed by *Klebsiella pneumoniae, Enterococci, Proteus mirabilis, Pseudomonas aeruginosa, Acinetobacter baumannii*, and fungal species like *Candida*.

*Klebsiella pneumoniae*, isolates screened as the cause of UTIs is a Gram-negative bacterium, that typically inhabits mucosal surfaces in animals and environmental sources like water and soil. In humans, it primarily colonizes the gastrointestinal tract and, to a lesser extent, the nasopharynx. From these sites, *K. pneumoniae* can disseminate into the blood or other tissues, leading to infections. Historically, before the discovery of antibiotics, *K. pneumoniae* played a significant role in community-acquired pneumonia (CAP), particularly affecting individuals with predisposing factors such as diabetes or alcoholism. After, the introduction of antibiotics, it transitioned into a prominent nosocomial pathogen, contributing to healthcare-associated infections. This shift underscores the adaptability of *K. pneumoniae* and its ability to exploit healthcare environments for transmission and infection (Wang et al., 2020; Schwenger et al., 2015).

*Klebsiella pneumoniae* ranks second to UPEC as a cause of nosocomial UTIs (8), and it is a significant opportunistic pathogen in immunocompromised individuals and hospitalized patients. The emergence of multidrug-resistant (MDR) strains of *K. pneumoniae* has posed considerable challenges in the treatment and management of UTIs caused by this bacterium (Clegg and Murphy, 2017). *K. pneumoniae* isolates are prone to developing resistance, often known for the production of extended-spectrum *β*-lactamases (ESBLs). These ESBL-producing strains of *K. pneumoniae* are now widespread globally, carbapenems are typically considered the first-line treatment for infections caused by ESBL-producing *K. pneumoniae* but later the bacterium quickly developed carbapenem resistance.

Carbapenem-resistant *K. pneumoniae* (CRKP) poses a significant threat due to their extended antibiotic-resistant profiles and global dissemination through mobile genetic elements. Notably, CRKP infections are associated with a higher mortality rate compared to carbapenem-susceptible strains (Podschun et al., 1998). Carbapenem resistance in *K. pneumoniae* arises through various mechanisms, including the production of Carbapenamase, loss or reduced expression of outer membrane proteins (OMPs), overexpression of *AmpC* cephalosporinases and ESBLs, and activation of efflux pumps. Among these, Carbapenamase production is the primary driver of carbapenem resistance. Understanding these resistance mechanisms is crucial for developing effective treatment strategies and infection control measures to combat the spread of CRKP in healthcare settings (Lan et al., 2021; Podschun et al., 1998; Xu et al., 2017).

Due to rising antimicrobial resistance among uropathogens, it is important to have local hospital-based knowledge of the pathogen-causing UTIs and their antibiotic sensitivity patterns. It is important to know the local antibiotic resistance patterns to implement suitable infection control measures and develop a rational antibiotic policy with local recommendations. These surveillance data are also used to assess the effectiveness of the measures taken and to identify new points for intervention to control bacterial resistance (Rizwan et al., 2018). The present study evaluated the antibiotic resistance pattern in the *K. pneumoniae* species over 10 years isolated in a tertiary care Hospital in Mysuru, South India.

## 2 Methodology

This retrospective study was conducted at an 1800-bed tertiary care hospital in Mysuru, South India, spanning January 2014 to December 2023 (10 years). Urine culture data from both outpatients and inpatients were extracted from the hospital information system (HIS). The extracted data included patient identifiers, hospital wards/outpatient departments, sample collection dates, specimen types, and results of speciation and antimicrobial susceptibility testing (AST). The study analyzed patients across different age and sex groups. Most of the specimens received were midstream urine samples, followed by catheterized samples and suprapubic aspirates.

Urine analysis was performed following standard microbiological procedures per Clinical and Laboratory Standards Institute (CLSI) guidelines, from urine microscopy to AST abbreviation already provided in previous paragraph so please remove that here (AST). A wet film examination of urine was conducted to detect inflammatory cells. Urine samples with significant bacteriuria were subjected to bacterial identification and AST using the automated VITEK-2 Compact system (bioMérieux, France). Gram-positive organisms were analyzed using P-628 cartridges, while Gram-negative organisms were assessed using N-405 cartridges for lactose fermenters and N-406 cartridges for non-lactose fermenters.

The clinical samples received by the laboratory for routine analysis were processed as per the standard protocols. Therefore, the study did not require oversight by the institutional ethics committee because of its descriptive nature.

### 2.1 Statistical analysis

Descriptive analysis was carried out to analyse the antimicrobial susceptibility pattern of *K. pneumoniae*. A Chi-square test for independence with a *p*-value <0.0001 was significant. Statistical analysis was performed using the Statistical Package for Social Sciences 20.0 program.

## 3 Results

The database revealed that, of the 73,283 urine samples received, 21,362 (29.15%) showed significant bacterial growth. The significant bacterial growth was highest in 2022, accounting for 31.43% (3,085/9,815), followed by 2020, accounting for 30.81% (2,227/7,227). The least significant growth was seen in 2014, accounting for 26.83% (1,348/5,024). The significant bacterial growth is given in Table 1.

**Table 1:** Significant bacterial growth over a decade.

Year	Total number of samples received	Samples with significant bacteriuria
2014	5,024	1,348 (26.83%)
2015	5,783	1,569 (27.13%)
2016	5,873	1,647 (28.04%)
2017	6,021	1,714 (28.46%)
2018	6,543	1,922 (29.37%)
2019	6,999	2,115 (30.21%)
2020	7,227	2,227 (30.81%)
2021	8,348	2,434 (29.15%)
2022	9,815	3,085 (31.43%)
2023	11,650	3,301 (28.33%)

### 3.1 Pathogens isolation rate from urine samples

**Figure 1:**
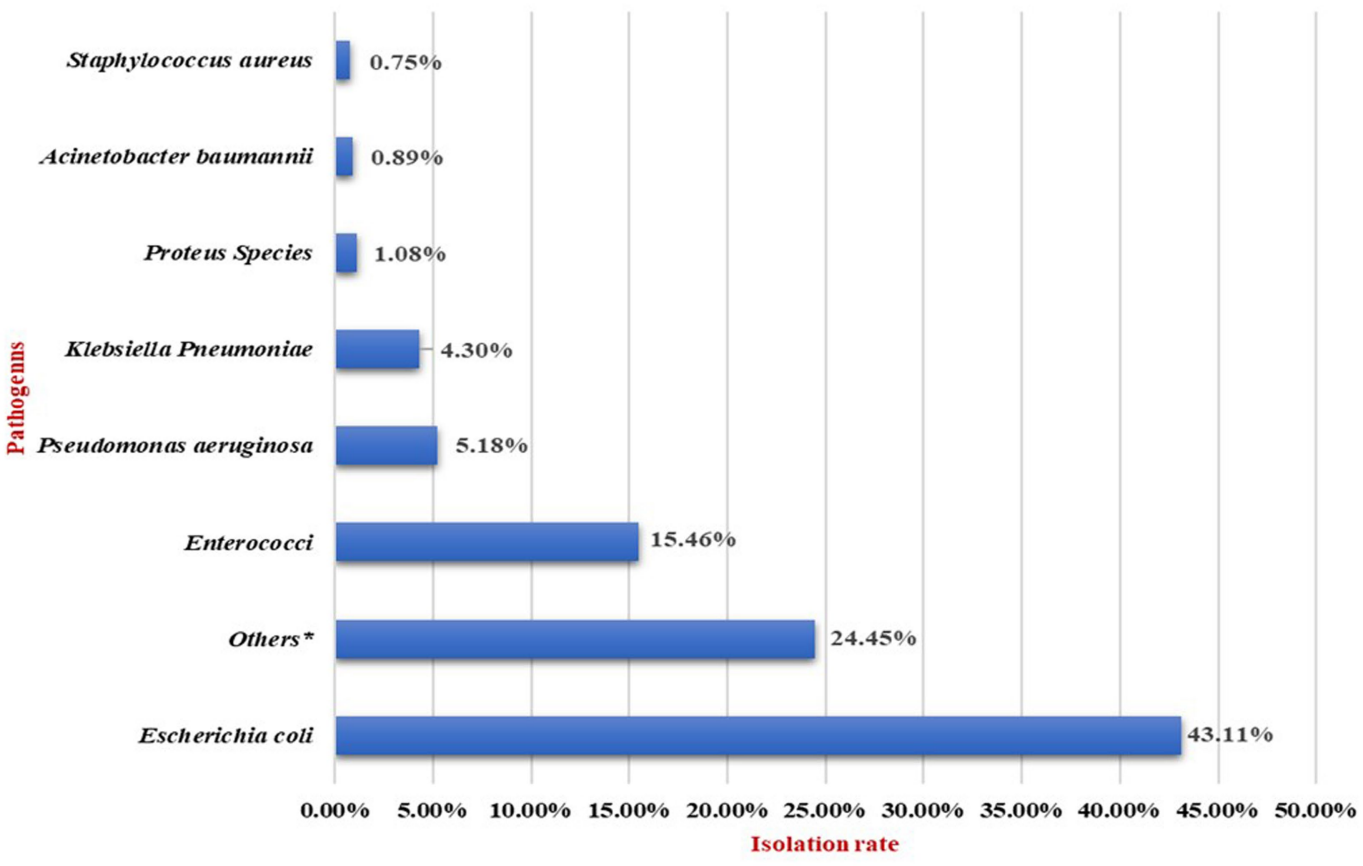
Uropathogens isolation rate over a decade.

In the 21,362 samples with significant growth, 21,434 distinct organisms were isolated. The increase in number of isolates is due to polymicrobial infection. Among the 21,434 distinct isolates, the majority of isolates were Gram-negative organisms followed by Gram-positive organisms. *E. coli* was the most frequently isolated pathogen, identified in 9,211 (43.11%) samples. This was followed by *Enterococci*, accounting for 3,313 (15.46%) of the total isolates; *P. aeruginosa*, 1,108 (5.18%); *K. pneumoniae*, 920 (4.30%); *Proteus* species, 232 (1.08%) and *A. baumannii,* 192 (0.89%). *S. aureus* was identified in 161 (0.75%) samples. The remaining 6,225 (24.45%) isolates included other pathogens, such as coagulase-negative *staphylococci* (CONS)*, Candida* species, and other Enterobacterales as shown in Figure 1.

### 3.2 *Klebsiella pneumoniae* isolation rate over 10 years

**Figure 2:**
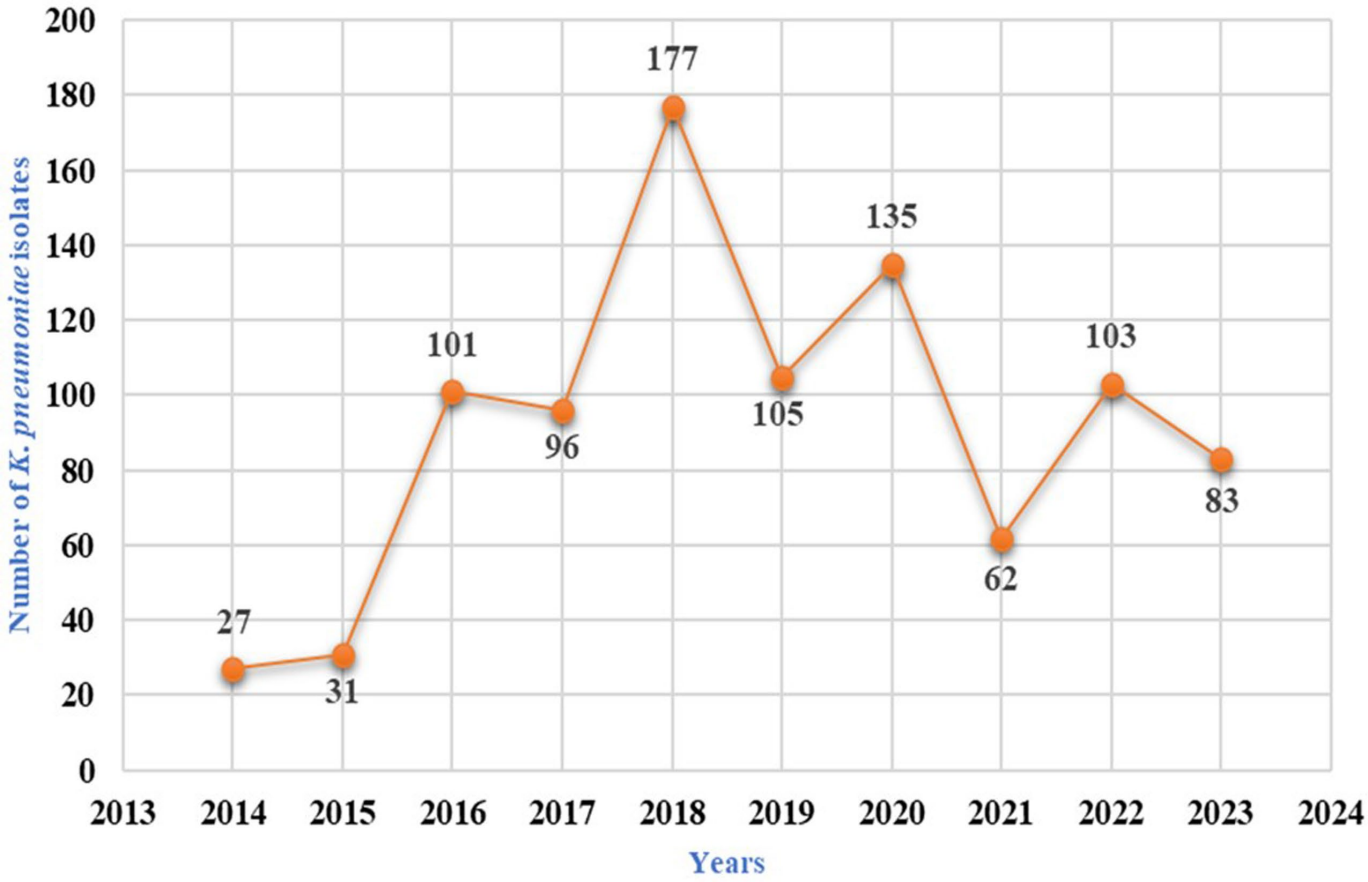
Klebsiella pneumoniae isolation rate over a decade from urine samples.

Over the decade, 920 *K. pneumoniae* isolates were identified, as given in Table 2. The highest number of *K. pneumoniae* were isolated in 2018, with 177 (9.21%) isolates. This was followed by 2020, with 135 (6.06%) isolates; 2019, with 105 (4.96%) isolates; 2022, with 103 (3.34%) isolates; 2016, with 101 (6.13%) isolates; 2017, with 96 (5.6%) isolates; 2023, with 83 (2.51%) isolates; 2021, with 62 (2.55%) isolates; 2015, with 31 (1.98%) isolates; and 2014, with 27 (2%) isolates. The trend of *K. pneumoniae* isolation rates over the decade are shown in Figure 2.

**Table 2:** Rate of *K. pneumoniae* among the total positive samples over the years.

Year	Number of positive cases of *Klebsiella pneumoniae* (*n* = 920)	Percentage of positive cases of *Klebsiella pneumoniae*	Total cases with significant growth (*n* = 21,362)
2014	27	2	1,348
2015	31	1.98	1,569
2016	101	6.13	1,647
2017	96	5.60	1714
2018	177	9.21	1922
2019	105	4.96	2,115
2020	135	6.06	2,227
2021	62	2.55	2,434
2022	103	3.34	3,085
2023	83	2.51	3,301

### 3.3 *Klebsiella pneumoniae* isolation frequency in male–female

Over the decade, the *K. pneumoniae* isolation frequency by gender revealed that *K. pneumoniae* had the highest incidence in females, with 519 isolates (56.41%), compared to 401 isolates (43.59%) in males.

### 3.4 Antimicrobial susceptibility pattern of *Klebsiella pneumoniae* over a decade

The antibiotic susceptibility profile of *K. pneumoniae* has shown a concerning trend of increasing resistance across multiple antibiotics over a decade. The detailed antimicrobial susceptibility pattern of *K. pneumoniae* is presented in Tables 3, 4. The Resistance to carbapenems increased dramatically from 3.70% (1/27 in 2014) to 66.13% (41/62 in 2021), with a subsequent decline to 30.10% (31/103) in 2022 and 38.55% (32/83) in 2023. Similarly, fluoroquinolone resistance rose from 11.11% (3/27 in 2014) to 87.10% (54/62 in 2021) and then to 42.72% (44/103) in 2022 followed by 40.96% (34/83) in 2023. Aminoglycoside resistance rose from 48.15% (13/27 in 2014) to 69.88% (58/83 in 2023). Resistance to amoxicillin/clavulanate increased from 11.11% (3/27 in 2014) to 86.75% (72/83 in 2023). Piperacillin/tazobactam resistance similarly increased from 51.85% (14/27 in 2014) to 86.75% (72/83). Resistance to cefoparazone/sulbactam rose from 29.03% (9/31 in 2015) to 62.65% (52/83 in 2023). Resistance to cotrimoxazole rose from 55.56% (15/27) in 2014 to 75.90% (63/83) in 2023. Various cephalosporins also showed increasing resistance: cefepime from 48.15% (13/27) in 2014 to 85.54% (71/83) in 2023, ceftriaxone from 7.41% (2/27) in 2014 to 89.16% (74/83) in 2023, cefuroxime from 37.04% (10/27) in 2014 to 85.54% (71/83) in 2023, and cefuroxime axetil from 3.70% (1/27) in 2014 to 91.57% (76/83) in 2023.

**Table 3:** Antimicrobial susceptibility pattern of *K. pneumoniae* from 2014 to 2018.

Antimicrobial agent	2014		2015		2016		2017		2018	
	S	R	S	R	S	R	S	R	S	R
Carbapenems	3.70%	96.30%	29.03%	70.97%	33.66%	66.34%	26.04%	73.96%	39.55%	60.45%
Fluroquinolones	51.85%	45.16%	45.16%	54.84%	50.50%	49.50%	57.29%	42.71%	59.89%	40.11%
Aminoglycosides	48.15%	51.85%	45.16%	54.84%	41.58%	58.42%	42.71%	57.29%	48.02%	51.98%
Amoxicillin/clavulanate	11.11%	88.89%	51.61%	48.39%	58.42%	41.58%	50.00%	50.00%	66.10%	33.90%
Piperacillin/tazobactam	51.85%	48.15%	45.16%	54.84%	47.52%	52.48%	48.96%	51.04%	61.58%	38.42%
Cefoparazone/sulbactam	0	100%	29.03%	70.97%	41.58%	58.42%	43.75%	56.25%	48.02%	51.98%
Trimethoprim/sulfamethoxazole	55.56%	44.44%	32.26%	67.74%	39.60%	60.40%	38.54%	61.46%	53.11%	46.89%
Cefepime	48.15%	51.85%	35.48%	64.52%	49.50%	50.50%	44.79%	55.21%	49.15%	50.85%
Ceftriaxone	7.41%	92.59%	51.61%	48.39%	61.39%	38.61%	47.92%	52.08%	62.71%	37.29%
Cefuroxime	37.04%	62.96%	45.16%	54.84%	63.37%	36.63%	50.00%	50.00%	62.15%	37.85%
Cefuroxime axetil	3.70%	96.30%	45.16%	54.84%	67.33%	32.67%	52.08%	47.92%	65.54%	34.46%

S, Sensitive; R, Resistant; %, Percentage. Carbapenems: include Ertapenem, Imipenem and Meropenem. Fluroquinolones: Ciprofloxacin.

**Table 4:** Antimicrobial susceptibility pattern of *K. pneumoniae* from 2019 to 2023.

Antimicrobial agent	2019		2020		2021		2022		2023	
	S	R	S	R	S	R	S	R	S	R
Carbapenems	53.33%	46.67%	63.70%	36.30%	66.13%	33.87%	30.10%	69.90%	38.55%	61.45%
Fluroquinolones	67.62%	32.38%	75.56%	24.44%	87.10%	12.90%	42.72%	57.28%	40.96%	59.04%
Aminoglycosides	59.05%	40.95%	63.70%	36.30%	67.74%	32.26%	57.28%	42.72%	69.88%	30.12%
Amoxicillin/clavulanate	65.71%	34.29%	81.48%	18.52%	80.65%	19.35%	36.89%	63.11%	86.75%	13.25%
Piperacillin/tazobactam	71.43%	28.57%	72.59%	27.41%	80.65%	19.35%	33.98%	66.02%	86.75%	13.25%
Cefoparazone/sulbactam	59.05%	40.95%	66.67%	33.33%	72.58%	27.42%	49.51%	50.49%	62.65%	37.35%
Trimethoprim/sulfamethoxazole	65.71%	34.29%	62.22%	37.78%	74.19%	25.81%	68.93%	31.07%	75.90%	24.10%
Cefepime	57.14%	42.86%	72.59%	27.41%	74.19%	25.81%	74.76%	25.24%	85.54%	14.46%
Ceftriaxone	73.33%	26.67%	78.52%	21.48%	87.10%	12.90%	78.64%	21.36%	89.16%	10.84%
Cefuroxime	67.62%	32.38%	73.33%	26.67%	80.65%	19.35%	86.41%	13.59%	85.54%	14.46%
Cefuroxime axetil	63.81%	36.19%	71.85%	28.15%	79.03%	20.97%	85.44%	14.56%	91.57%	8.43%

S, Sensitive; R, Resistant; %, Percentage. Carbapenems: include Ertapenem, Imipenem and Meropenem. Fluroquinolones: Ciprofloxacin.

### 3.5 Rate of carbapenem-resistant *Klebsiella pneumoniae* over 10 years

**Figure 3:**
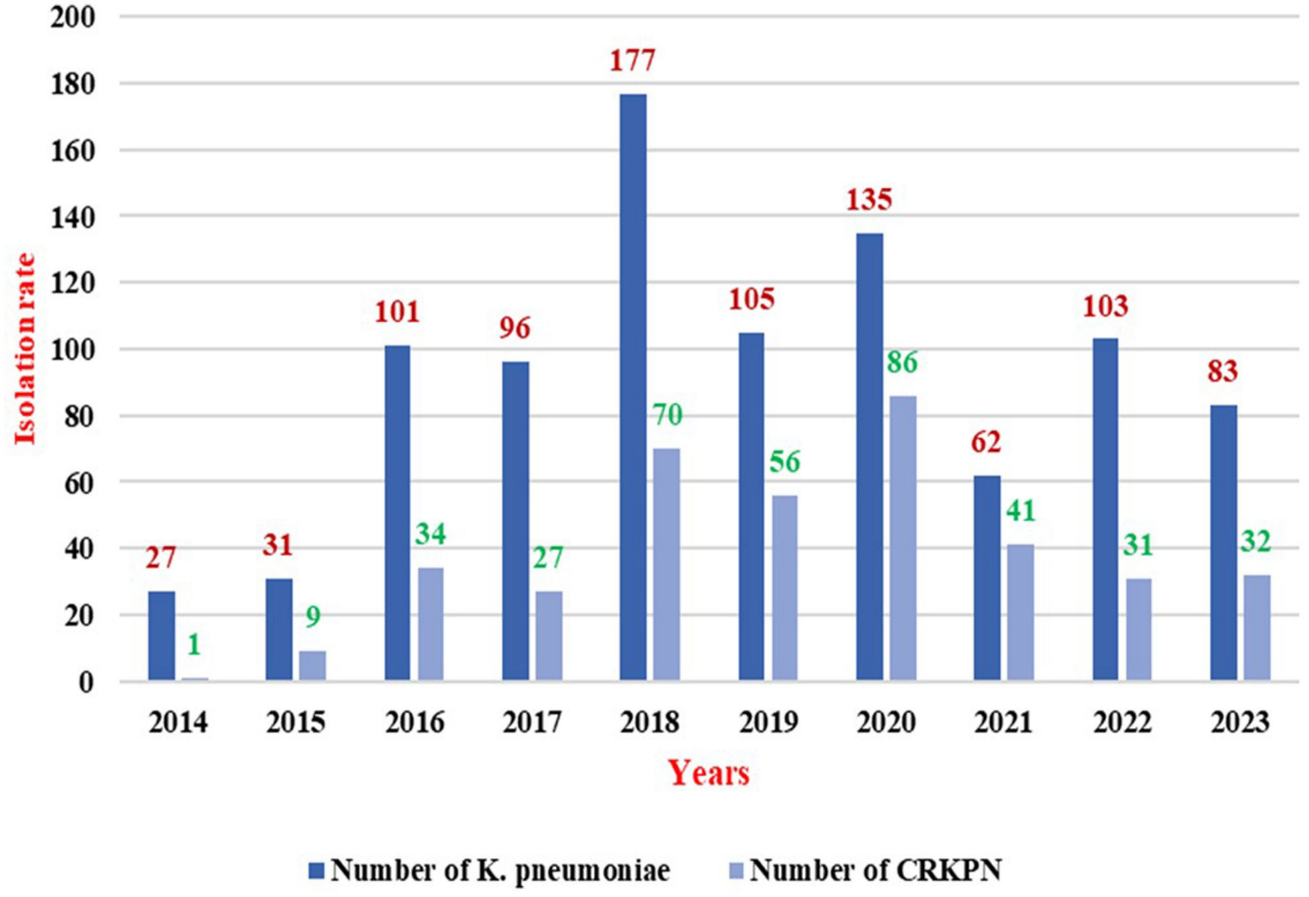
Carbapenem resistant Klebsiella pneumoniae isolation rate over a decade.

Over the decade, 920 *K. pneumoniae* isolates were identified, of which 385 (41.84%) were resistant to carbapenems. As represented in Figure 3, the resistance rates over the years. In 2014, resistance was observed in 3.70% (1/27) of the isolates, which increased to 29.03% (9/31) in 2015. The resistance rate continued to rise in 2016, reaching 33.66% (34/101), then slightly decreasing to 26.04% (25/96) in 2017. In 2018, resistance increased to 39.55% (70/177), followed by a further increase to 53.33% (56/105) in 2019 and 63.70% (86/135) in 2020. By 2021, the resistance rate reached its highest point at 66.13% (41/62). However, a decline was observed in 2022, with resistance decreasing to 30.10% (31/103) and stabilizing at 38.55% (32/83) in 2023.

### 3.6 Carbapenem-resistant *Klebsiella pneumoniae* isolation trend in male and females

**Figure 4:**
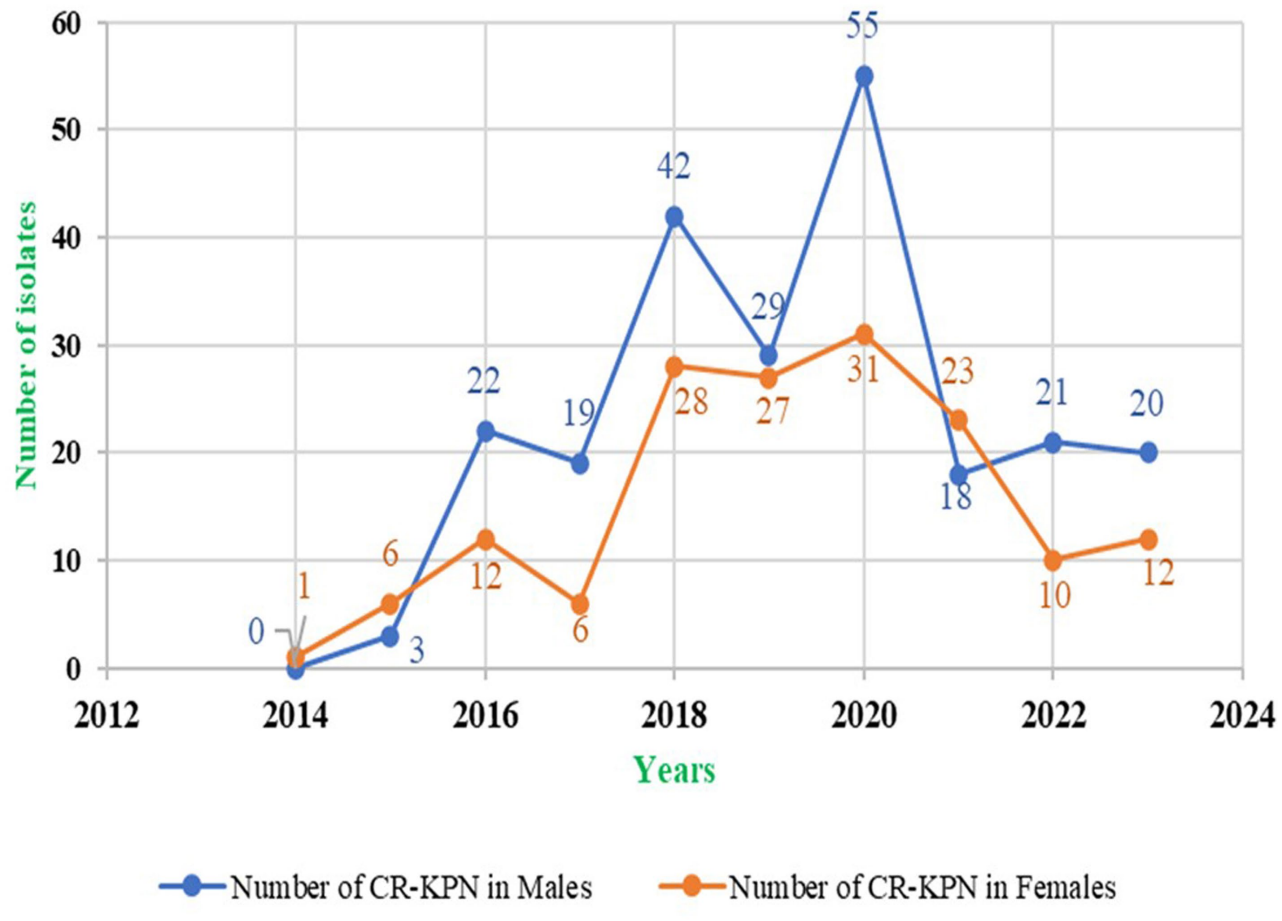
Carbapenem resistant Klebsiella pneumonae isolation trend in males and females over a decade.

As presented in Table 5, over the decade, the rate of carbapenem-resistant *K. pneumoniae* (CRKP) isolation was higher in males, accounting for 229 (59.48%) isolates, compared to females, who accounted for 156 (40.52%) isolates. However, in 2015, the CRKP isolation rate was higher in females, accounting for 66.67% (6/9) of the isolates, and in 2022, females again accounted for a higher proportion, representing 56.10% (23/41) of the CRKP isolates. To calculate the *p*-value for the comparison of the proportions of CRKP isolation in males and females over the decade, the Chi-square test for independence or Fisher’s exact test was used. The *p* value was extremely small (*p* < 0.0001), indicating a statistically significant difference in the distribution of CRKP isolates between the two genders. The CRKP isolation trend in males and females is shown in Figure 4.

**Table 5:** The male–female ratio in carbapenem-resistant *K. pneumoniae* over a decade.

Year	CR-KPN numbers in Male	CR-KPN percentage in Males	CR-KPN numbers in females	CR-KPN percentage in females	Total no of CR-KPN	*p*-value
2014	0	0.00	1	100	1	<0.0001
2015	3	33.33	6	66.67	9	<0.0001
2016	22	64.71	12	35.29	34	<0.0001
2017	19	76.00	6	24.00	25	<0.0001
2018	42	60.00	28	40.00	70	<0.0001
2019	29	51.79	27	48.21	56	<0.0001
2020	55	63.95	31	36.05	86	<0.0001
2021	18	43.90	23	56.10	41	<0.0001
2022	21	67.74	10	32.26	31	<0.0001
2023	20	62.50	12	37.50	32	<0.0001
Total	229	59.48	156	40.52	385	<0.0001

CR-KPN, Carbapenem resistant *K. pneumoniae*.

## 4 Discussion

This retrospective study aimed to evaluate the prevalence and antimicrobial susceptibility patterns of *K. pneumoniae* isolated from UTI patients at a tertiary care hospital in South India. Over 10 years (2014–2023), the laboratory processed 73,283 urine samples, among them 21,362 samples yielded significant bacterial growth, yielding an overall positivity rate of 29.15%. These findings align with those of similar studies. Jayaprakash and Bai (2016) reported a positivity rate of 34.73% (191/550) in urine samples. Similarly, Chatterjee et al. (2016) documented significant bacteriuria in 35.9% of patients over 1 year in Eastern India. In contrast, Janifer et al. (2009) observed significant growth in 42.8% (496/1,157) of participants over 1 year in South India. Notably, no other studies report data spanning a decade; hence, the authors compared their findings with studies conducted over shorter durations. In the Western literature, Zhang et al. (2022) isolated 6,000 organisms from 20,000 urine samples (30%) while Smith et al. (2020) reported 2,700 organisms from 10,000 urine samples (27%) over 8 years. Thompson et al. (2022) identified 3,200 organisms from 8,500 urine samples (37.65%) over 5 years.

The consistent urine culture positivity rate of 30% across multiple studies can be attributed to standardized diagnostic criteria, stable uropathogen prevalence, and clinical indications for culture testing. Our study revealed that common pathogen isolated accounting for 9,211 (43.11%) of the isolates, followed by *Enterococci* for 4,313 (20.19%) of the isolates, *P. aeruginosa* for 1,108 (5.18%) of the isolates, *K. pneumoniae* for 920 (4.3%) of the isolates, *A. baumannii* for 192 (0.89%) of the isolates, *Proteus* species for 232 (1.08%) of the isolates and *S. aureus* for 161 (0.75%) of the isolates.

In a study by Bhargava et al. (2022) in India over 1 year, *E. coli* was the most prevalent pathogen at 55.0%, followed by *Proteus species* (6.9%), *K. pneumoniae* (6.6%), and *P. aeruginosa* (6.3%). In a study by Dash et al. (2013), conducted in 2013 in Odisha, India, *E. coli* was the most common pathogen, accounting for 68.8% of isolates, with *Enterococcus species* at 9.7%. Similarly, a study by Tilahun et al. (2024), conducted in 2023 in northeast Ethiopia over 10-month study, found that out of 346 participants, 92 (26.6%) were culture positive. Of these, 75 (81.52%) were bacterial and 17 (18.48%) were fungal. The most frequently isolated bacteria were *E. coli* (16/75, 21.33%), followed by *K. pneumoniae* (11/75, 14.67%) and *Enterococcus species* (10/75, 10.87%) (Tilahun et al., 2024).

This dominance of *E. coli* across multiple studies can be attributed to its array of virulence factors, including adhesins such as fimH, which facilitate urothelial attachment, biofilm formation, and antibiotic resistance mechanisms (Johnson and Russo, 2002; Klein and Hultgren, 2020). The high prevalence of *E. coli* in UTIs can also be linked to its ability to colonize the gastrointestinal and urogenital tracts, making it a primary cause of both community-associated UTIs (CA-UTIs) and hospital-acquired UTIs (HA-UTIs). CA-UTIs often result from ascending infections originating from perineal flora, while HA-UTIs are frequently associated with catheterization and prolonged hospital stays, which provide an environment conducive to biofilm formation and antibiotic resistance (Johnson et al., 2022). These data also imply that the most common etiological agents of UTIs are gram-negative organisms, with gram-positive organisms closely behind. This predominance of gram-negative pathogens, particularly *E. coli* and *K. pneumoniae*, can be attributed to their structural advantages, such as an outer membrane that enhances antibiotic resistance and virulence factors that promote persistence in the urinary tract (Johnson and Russo, 2002). Additionally, the widespread use of broad-spectrum antibiotics has led to the selection of multidrug-resistant gram-negative strains, contributing to their increasing prevalence in both CA-UTIs and HA-UTIs (Martinez et al., 2021).

The antibiotic susceptibility profiles of *K. pneumoniae* revealed a concerning trend of increasing resistance across multiple antibiotics. Over 10 years, resistance to carbapenems increased sharply from 51.85% (14/27) in 2014 to 66.13% (41/62) in 2021. Interestingly, the resistance then decreased to 30.10% (31/103), followed by 38.55% (32/83) in 2022 and 2023. Similarly, the Fluoroquinolone resistance increased from 11.11% (3/27) in 2014 to 87.10% (54/62) in 2021 and later decreased to 42.72% (44/103) in 2022, followed by 40.96% (34/83) in 2023. Aminoglycoside resistance increased from 48.15% (13/27) in 2014 to 69.88% (58/83) in 2023, and in amoxicillin/clavulanate resistance increased from 11.11% (3/27) in 2014 to 86.75% (72/83) in 2023. Resistance to piperacillin/tazobactam increased from 51.85% (14/27) in 2014 to 86.75% (11/83) in 2023, while in cefoparazone/sulbactam, resistance increased from 29.03% (9/31) in 2014 to 62.65% (52/83) in 2023. Cotrimoxazole resistance rose from 55.56% (15/27) in 2014 to 75.90% (63/83) in 2023. Cephalosporins also showed increased resistance: cefepime from 48.15% (13/27) in 2014 to 85.54% (71/83) in 2023, ceftriaxone from 7.41% (2/27) in 2014 to 89.16% (74/83) in 2023, cefuroxime from 37.04% (10/27) in 2014 to 85.54% (71/83) in 2023, and cefuroxime Axetil from 3.70% (1/27) in 2014 to 91.57% (76/83) in 2023.

In a study conducted by Sharma et al. (2016) in India over 3 years, *K. pneumoniae* isolated from urine samples exhibited high resistance rates, with 72.71% resistance to fluoroquinolones and 76.22% resistance to aminoglycosides on average. Similarly, a study by Grover et al. (2004) in New Delhi conducted for 1 year found resistance rates for Uropathogenic *K. pneumoniae* varied from 39.2 to 88.0% for cephalosporins (including cefoxitin, cefuroxime, cefotaxime, ceftazidime, and cefepime) and from 51.0 to 90.2% for non-cephalosporins (such as aztreonam, piperacillin, chloramphenicol, and trimethoprim-sulfamethoxazole). Globally, there is an escalating trend of antibiotic resistance in *K. pneumoniae*. The prevalence of multidrug-resistant strains of *K. pneumoniae* in hospital-acquired infections is a growing concern observed in both Europe and Asia (Santana et al., 2016). A study conducted by Ndip et al. (2001) in Cameroon revealed seven distinct anti-biotypes of *Klebsiella pneumoniae* isolated from urine, while all isolates showed resistance to amoxicillin and trimethoprim, they displayed notable susceptibility to norfloxacin (90.01%), cefuroxime (95.45%), and ciprofloxacin (86.36%). In a 2022 study by Della Rocca et al. (2022) in Italy over 5 years, more than 50% of isolates from urine samples were resistant to cephalosporins, fluoroquinolones, and penicillin. Resistance to carbapenems and aminoglycosides was lower, ranging between 20 and 40% (Della Rocca et al., 2022). Another study by Zhang et al. (2021) in China (2018), conducted for 3 years starting in 2014, showed that in medical ICUs, resistance rates for UTI-derived *K. pneumoniae* isolates increased to 50–60% for amikacin, imipenem, and ertapenem by 2017.

This increase can be attributed to multiple factors, including the overuse and misuse of these antibiotics in both community and healthcare settings (Patel et al., 2023). Additionally, molecular epidemiological studies have identified the emergence of resistant clones, such as extended-spectrum beta-lactamase (ESBL)-producing *E. coli* and carbapenem-resistant *K. pneumoniae*, which have played a crucial role in this trend (Lee et al., 2021). The rapid spread of mobile genetic elements, including plasmids encoding beta-lactamase enzymes, has further exacerbated the resistance crisis, making empirical treatment increasingly challenging.

Carbapenem, a potent antibiotic to treat infections caused by Gram-negative bacteria, has been used for a long time as a first choice to treat infections caused by ESBL-producing bacteria. However, resistance to carbapenems is alarmingly increasing. The rapid increase in carbapenem-resistant Enterobacteriaceae (CRE) has been reported both locally and globally (Papp-Wallace et al., 2011). In our study, carbapenem resistance in *K. pneumoniae* surged dramatically from 3.70% (1/27) in 2014 to 66.13% (41/62) in 2021 but then saw a gradual decline to 30.10% (31/103) in 2022 and 38.55% (32/83) in 2023. Similarly, fluoroquinolone resistance increased from 11.11% (3/27) in 2014 to 87.10% (54/62) in 2021 and decreased to 42.72% (44/103) and 40.96% (34/83) in 2022 and 2023. Supporting these findings, a 10-year study by Liao et al. (2023) (2011–2020) in the United States documented a decrease in carbapenem resistance in *Klebsiella pneumoniae* from 10.6% in 2011 to 1.5% in 2020, and a similar trend for fluoroquinolone resistance, which fell from 18% in 2011 to 11.7% in 2020, dropping below the 20% threshold. This reduction indicates improved availability of empiric antibiotic options during this period (Liao et al., 2023). Additionally, research by Chang et al. (2023) in Southwest China from 2018 to 2022 revealed a steady decrease in carbapenem resistance rates among *K. pneumoniae* isolates. Specifically, resistance to imipenem and meropenem fell from 14.5 and 17.8% to 14.0 and 14.4%, respectively. This overall reduction in drug resistance can be attributed to two main factors. Firstly, national policies have successfully institutionalized antimicrobial drug management, enhanced medical professionals’ understanding of appropriate antimicrobial use, and increased public awareness of drug resistance issues. Secondly, improvements in bacterial resistance monitoring networks have provided valuable data for adjusting management policies and guiding medical institutions in selecting effective antibiotics. Ceftazidime/avibactam, tigecycline, and polymyxin are now considered among the most effective treatments for infections caused by carbapenem-resistant Gram-negative bacteria (CRGNB) (Chang et al., 2023).

In our study, the prevalence of carbapenem-resistant *K. pneumoniae* was significantly higher in males (59%, 229/385) compared to females (41%, 156/385). This contrasts with findings from a 2020 study by Liu et al. (2020) which reported that over half of patients with UTI were male (60%) over nearly 5 years. This difference may be attributed to factors such as age-related or functional urethral obstruction, which necessitates catheterization and alters the microbial environment of the urine. This change can lead to biofilm formation and the development of drug-resistant bacteria (Liu et al., 2020). Additionally, a study by Park et al. (2022), covering 2 years (2018–2020) in Korea, found that men under 80 years of age had a higher infection rate with *K. pneumoniae* compared to women. This lower prevalence in women might be explained by better adherence to hygiene practices among women, such as hand hygiene and soap use, as suggested by community studies (Park et al., 2022).

In a study by Halim et al. (2020) conducted in North India, in 2021, a high carbapenem resistance rate of 55.2% was reported in *K. pneumoniae* isolates from urine samples. In a 2021 study conducted in Tamil Nadu, India, Sureka Indrajith et al. discovered that 58% of *K. pneumoniae* strains were resistant to carbapenems over 1 year in urine samples (Indrajith et al., 2021). A decade-long study by Jalal et al. in Saudi Arabia, published in 2023, highlighted a dramatic rise in carbapenem resistance to *K. pneumoniae*, increasing from 6.6% in 2011 to 59.9% in 2021, with a higher prevalence in male patients compared to females (Jalal et al., 2023). Additionally, a 2020 study by Yanyan Hu et al. in China documented a significant increase in carbapenem resistance in *Klebsiella pneumoniae*, rising from 2.5 to 15.8% over the decade from 2008 to 2018, with 55% of the resistant cases involving male patients (Hu et al., 2020).

Carbapenems are the first-line drugs for the treatment of infections caused by multidrug-resistant bacteria like *Klebsiella pneumoniae*. Carbapenem resistance arises from various mechanisms, including reduced antibiotic entry via porin channels (Omp) and active efflux pumps that expel carbapenems before they act. Hydrolytic modification by *β*-lactamases is another critical resistance mechanism, with metallo-*β*-lactamases (MBLs) being notable for hydrolyzing carbapenems. These enzymes render many *β*-lactam antibiotics ineffective. *β*-lactamases are classified into four groups based on amino acid sequences: A, B, C, and D, and are encoded on chromosomes (Aurilio et al., 2022; Alizadeh et al., 2020).

Class A includes extended-spectrum *β*-lactamases (ESBLs) and *Klebsiella pneumoniae* carbapenemases, while Class B includes MBLs. Class C enzymes (cephalosporinases) are resistant to cephamycins, and Class D enzymes (oxacillinases) hydrolyze carbapenems, with OXA-48 variants. The rise of ESBL-producing bacteria has increased carbapenem use, accelerating the emergence of carbapenemases, categorized into Ambler classes A, B, C, and D. Class A carbapenemases may be chromosomally encoded (e.g., SME, NmcA), plasmid-encoded (e.g., KPC, GES), or both (e.g., IMI). Class B MBLs are Verona integron-encoded MBL (VIM), imipenemase (IMP), and New Delhi MBL (NDM). Class D carbapenemases, like OXA-48. Although Class C enzymes have low carbapenemase activity, combined with reduced membrane permeability or efflux pump overexpression, they can contribute to resistance (Aurilio et al., 2022; Alizadeh et al., 2020).

These studies highlight the varied and concerning patterns of antibiotic resistance in *K. pneumoniae* across different regions, emphasizing the necessity for improved surveillance and targeted antibiotic stewardship programs to effectively manage the growing resistance in these isolates. This retrospective study underscores the increasing resistance in *K. pneumoniae*. The clinical efficacy of broad-spectrum antibiotics is jeopardized by their irrational use, emphasizing the urgent need for prudent antibiotic prescription practices, promoting antimicrobial stewardship programs, and enhancing infection control practices.

### 4.1 Strengths of the study

The retrospective study reveals the results of a large number of isolates collected over 10 years, which is a potential strength of the study.

### 4.2 Limitations of the study

Most of the *Klebsiella* isolates were obtained from complicated UTI cases, as they were received from the Urology and Nephrology departments. Therefore, the findings may not necessarily reflect the antibiogram of uncomplicated UTI cases. Additionally, in this study, we did not classify the cases based on their diagnoses. Authors could not classify them according to age group, type of urine specimen and type of diagnosis.

## 5 Conclusion

This study highlights the significant rise in antimicrobial resistance among *Klebsiella pneumoniae-*causing UTIs. High levels of resistance to antibiotics, including carbapenems and fluoroquinolones, pose significant challenges in the treatment and management of these infections. Effective management requires tailored antibiotic stewardship initiatives and strict infection control measures. Continuous surveillance of resistance patterns is essential to guide appropriate antibiotic use and preserve treatment options in clinical practice.

## Data Availability

The original contributions presented in the study are included in the article/supplementary material, further inquiries can be directed to the corresponding author.
